# Socioeconomic per-case costs of stroke, myocardial infarction, and preterm birth attributable to air pollution in Sweden

**DOI:** 10.1371/journal.pone.0290766

**Published:** 2024-01-11

**Authors:** Hedi Katre Kriit, Johan Nilsson Sommar, Stefan Åström

**Affiliations:** 1 Department of Public Health and Clinical Medicine, Section of Sustainable Health, Umeå University, Umeå, Sweden; 2 Institute of Global Health, Health Economic and Financing Group, Heidelberg University, Heidelberg, Germany; 3 Interdisciplinary Center for Scientific Computing, Climate-Sensitive Infectious Disease Lab, Heidelberg University, Heidelberg, Germany; 4 Anthesis, Gothenburg, Sweden; University of the Witwatersrand Johannesburg, SOUTH AFRICA

## Abstract

**Background:**

Incident cases of stroke, myocardial infarction, and preterm birth have established exposure-response functions associated with air pollution. However, there are no studies reporting detailed costs per case for these health outcomes that are adapted to the cost-benefit tools that guide the regulation of air pollution.

**Objectives:**

The primary objective was to establish non-fatal per-case monetary estimates for stroke, myocardial infarction, and preterm birth attributable to air pollution in Sweden, and the secondary objective was to ease the economic evaluation process of air pollution morbidity effects and their inclusion in cost-benefit assessments.

**Methods:**

Based on recommendations from the literature, the case-cost analysis considered direct and indirect medical costs, as well as production losses and informal costs relevant for the calculation of the net present value. A literature search was conducted to estimate the costs of each category for each incident case in Sweden. Informal costs were estimated using the quality-adjusted life-years approach and the corresponding willingness-to-pay in the Swedish population. The total average per-case cost was estimated based on specific health outcome durations and severity and was discounted by 3.5% per year. Sensitivity analysis included varying discount rates, severity of health outcome, and the range of societal willingness to pay for quality-adjusted life years.

**Results:**

The average net present value cost estimate was €_2016_ 460k (185k–1M) for non-fatal stroke, €_2016_ 24k (16k–38k) for myocardial infarction, and €_2016_ 34k (19k–57k) for late preterm birth. The main drivers of the per-case total cost estimates were health outcome severity and societal willingness to pay for risk reduction. Varying the discount rate had the largest effect on preterm birth, with costs changing by ±30% for the discount rates analysed.

**Recommendation:**

Because stroke, myocardial infarction, and preterm birth have established exposure-response functions linking these to air pollution, cost-benefit analyses should include the costs for these health outcomes in order to adequately guide future air pollution and climate change policies.

## Introduction

Air pollution is the number one environmental risk factor for human health according to the World Health Organization (WHO) [1, 2], and an increasing number of epidemiological studies have linked several chronic health outcomes with high air pollution concentrations [3]. Health outcomes such as cardiovascular diseases and preterm birth (PTB) have established exposure-response functions (ERFs) with regards to exposure to particulate matter with an aerodynamic diameter less than 2.5 μm (PM_2.5_) [4, 5], including stroke and myocardial infarction (MI) with estimated risk increases of 19% and 13%, respectively, for each 10 μg/m^3^ increase in long-term exposure to PM_2.5_ [5]. For PTB, a meta-analysis by Klepac et al. (2018) reported a 24% increase in risk for each 10 μg/m^3^ increase in PM_2.5_ [6].

Despite the high prevalence and increasing trend of cardiovascular disease burden and PTB [7, 8], currently available health economic assessments of air pollution include only a narrow range of related health outcomes [9]. Important international air pollution policy support tools providing information on the socio-economic benefits of reducing air pollution, such as BenMap used by the Environmental Protection Agency [10] in the US and Alpha Riskpoll (ARP) [11] used by the European Commission, need to be continuously updated as new epidemiological knowledge is developed. Such updates are needed because cost-benefit analysis (CBA) is becoming more important for air pollution policy. As an example, in 2016 the EU air pollution policy goals for 2030 were decided with a large influence from the European Commissions impact assessment (SWD (2013) 531), which was made with a CBA that had not been updated with the latest epidemiological knowledge. Together, the use of outdated data and the omission of new knowledge risk leading to suboptimal decisions regarding policies that are meant to guide development towards sustainability in the coming decades.

Both ARP and BenMap include ERFs for morbidity effects and mortality, where mortality currently constitutes 90% of the monetized benefits of reduced air pollution [10]. However, the short-term morbidity effects included in the CBAs rely on outdated ERFs from the 1980s or 1990s, such as restricted activity days and respiratory hospital admissions [12], and are currently omitting new knowledge from systematic reviews and medical expert guidelines on long-term chronic health outcomes such as cardiovascular disease, chronic obstructive pulmonary disease, and PTBs that are attributable to PM_2.5_ [3, 13], all which have profound long-term economic impacts both for the individual and for society. For example, the burden of disease for stroke and MI is estimated to correspond on average to 8% of all European health care expenditures [7]. Thus, for air pollution policy processes it is imperative to first value the socioeconomic cost per case in monetary terms and then to upgrade the economic assessments of air pollution policies.

The available literature presenting long term economic costs of these health outcomes is scarce and varies in terms of costs included and if they are disaggregated based on the severity of the condition. Below we present an overview of the economic literature for costs of stroke, MI, and PTB using monetary units and converted into €_2016_ using purchase power parity exchange rates and consumer price indices presented by the OECD to ease the comparison.

The costs for the health care sector of cardiovascular disease in Europe are estimated to be €_2016_ 111 billion annually, of which €_2016_ 45 billion is attributable to stroke burden [7]. For Sweden, the average lifetime cost per stroke case has been estimated to be €_2016_ 94k, while the distribution of costs can vary by up to six-fold due to patient-specific characteristics and the severity of stroke-induced disability [14]. A systematic review of MI costs reported mean direct costs to be €_2016_ 9k per incidence [15]; however, individual studies in Sweden and in Canada report higher mean direct costs for MI of €_2016_ 12–16k [16–18]. The productivity losses out of the total cost of stroke and MI have been estimated to be approximately 9% and 25%, respectively [14, 16]. In contrast, the cost of PTB has been estimated to be €_2016_ 290k per case, where productivity losses contribute about 85% [19]. However, a systematic review of PTB costs concluded that the existing literature omits several economic costs and that none of the health outcomes of interest in cost-of-illness studies have included the informal costs reflecting decreased quality of life [20]. Further, none of the published stroke and MI cost estimates have clearly separate costs for non-fatal cases and fatal cases, and the published PTB cost estimates do not separate costs according to gestational age. The separation of costs between fatal and non-fatal is of importance when assessing the air pollution health economic burden in order to avoid double counting because mortality-related health economic burden currently dominates.

The main aim of this study was to present the monetary per-case cost estimates of non-fatal stroke, MI, and PTB attributable to air pollution in Sweden. The secondary objective was to ease the economic evaluation process of air pollution morbidity effects and their inclusion in the cost-benefit assessments. This study was limited stroke, MI, and PTB because these are health effects for which there is a consensus in the epidemiological evidence.

## Materials and methods

### Literature search

To obtain the economic costs of stroke and MI attributable to air pollution, a literature search was conducted using the *PubMed* and *Google Scholar* search engines. The search word combinations were based on a previously published scientific paper reporting health care costs attributable to air pollution in the UK [21]. The search combination was extended to also include societal costs. The inclusion criteria were a) published in 2005–2020, b) using data for Sweden, c) outcomes classified according to ICD-code, d) costs representing the health outcome in question; and e) reporting both direct and indirect costs of the health outcome of interest. If more than one paper was found to fulfil all criteria for the same outcome, the most recent paper was chosen. For specific search combinations and the numbers of studies found and excluded, please see S1 File.

### Calculation of the Net Present Value (NPV) of specific outcomes

The monetization of impacts into economic costs was based on the method suggested by Hodek et al. [22], which is also the method recommended by Hunt et al. (2016) [9]. The economic cost categories considered both formal costs (direct medical costs (DMCs), direct non-medical costs (DNMCs), and indirect costs (ICs)) and informal costs (InCs) (Table 1) [22]. The data availability and aggregation level in the source literature determined which categories and subcategories were ultimately used in the NPV calculations.

**Table 1:** Cost categories for valuing the health outcomes and the available costs used for the monetization of stroke, myocardial infarction, and preterm birth.

	MI [16]	Stroke [23]	PTB
**DMC**			
Outpatient			
• visits to physicians (general practitioners and specialists)	X	X	X
• visits to non-physicians	NA	NA	NA
• medication	X	X	X
• aids and devices	X	X	X
Outpatient/home care	X	X	X
Inpatient			
• initial hospitalization	X	X	X
• re-hospitalization	X	X	X
• rehabilitation/regimen	NA	X	NA
**DNMC**			
• transportation	NA	NA	NA
• accommodation	NA	NA	NA
• home or car modifications	NA	X	NA
• meals	NA		NA
• other/special medical approaches	NA	X	NA
• childcare/babysitting for other siblings	NA	NA	NA
• special education/schooling	NA	NA	NA
• home help	NA	X	NA
**Indirect costs**			
• income losses	X	X	X
• missed working days	X	X	X
• time losses (opportunity costs)	NA	X	NA
**Informal costs**			
• Quality of Life	X	X	X
• Quality of Life of parents or other caregivers	NA	X	NA

*Abbreviations indicate for which outcome the cost item has been assessed. ST = stroke, MI = myocardial infarction, PTB = preterm birth, NA = not assessed.

Lifetime costs of the chronic health outcomes were calculated as the sum of the NPVs of the economic value of all cost categories and/or sub-categories (Eq 1). Therefore, the time of occurrence and duration of each sub-category also needed to be specified.

$$NPV_{cat}=\overset{T}{\underset{t=t_{inc}}{\sum }}\frac{c_{cat,t}}{{\left(1+r\right)}^{\left(t-t_{inc}\right)}}$$ (1)

Where:

NPV = Net Present Value

*t*_*inc*_ = life-year when incidence occurs

*T* = years of life expectancy

*r* = annual discount rate

*c*_*cat*,*t*_ = cost category or sub-category

For the calculation of the NPV we used an *r*-value of 3.5% (low 2%; high 5%) [24] and a *T* of 82 years (corresponding to the average life expectancy in Sweden [25]).

All DMCs, DNMCs, and ICs stated in the literature were adjusted for inflation between publication year and 2016 and then converted from the stated currency to €_2016_ using Purchase Power Parity-corrected exchange rates.

In impact assessments of air pollution policies, air pollution-induced mortality is often represented as ‘all-cause’ mortality. This caused a challenge for this study because stroke and MI can be fatal. To avoid double-counting in impact assessments and CBAs on air pollution policies, it is therefore important to separate the costs of fatal stroke and MI incidences from the costs of non-fatal incidences. To ensure this, we only made cost calculations for incidences where the patient survived >30 days after the stroke or MI incidence.

We used the degree of disability severity ranges reported from the source literature as input to calculate the uncertainty intervals of the cost estimates. For stroke, the disease severity classification based on the Modified Ranking Scale (mRS) was used to calculate low, middle, and high cost estimates. For MI, when no information on severity range was given we used a ±20% uncertainty range based on recommendations by Briggs et al. 2012 [26]. For the DMCs for PTB, we also used a ±20% uncertainty range. For PTB income losses we used varying gestational ages as inputs to compare low and middle cost estimates. For comparing middle and high cost estimates of PTB we used alternative sources for costs of work absenteeism expressed as productivity loss. The uncertainty ranges are presented within brackets in the subsequent parts of the method chapter.

### Formal costs

#### Stroke

Stroke survivors suffer varying degrees of permanent functional disability due to neurological damage. Thus, it is appropriate to assume that costs related to functional disability as a direct cause of stroke continue during the remaining lifetime after the incident. For two years, stroke patients identified with ischemic stroke (ICD-10 I63) and intracerebral haemorrhagic stroke (ICD-10 I61) were followed regarding their health care sector resource use by Lekander et al. [23]. For the DMC category, the inpatient costs in Lekander et al. [23] were highest during the first year of disease occurrence and showed a steep decrease during the second year among all groups stratified according to the degree of disability based on mRS. The DNMCs and ICs related to stroke were relatively small during the first year and rose considerably during the second year for all disability strata.

The results in Lekander et al. [23] were presented separately for ischemic and haemorrhagic stroke and stratified based on the degree of disability using the mRS, and the mRS categorization was used as the basis to categorize the uncertainty intervals. In the present study we weighted the costs for ischemic and haemorrhagic stroke in accordance with their relative share of cases in Lekander et al. [23] and used the average costs for ‘all survivors’ in the mRS strata to reflect the mid-range cost estimates, and low cost estimates corresponded to patients ranked with low disability (mRS 0–2) and high costs corresponded to patients with high disability (mRS 5) [23]. To calculate the stroke-related lifetime costs, we calculated the expected remaining life years for ischemic and intracerebral haemorrhagic stroke survivors in relation to average life expectancy *T* in Sweden and the *t*_*inc*_ values reported by Lekander et al. [23]. Because we obtained data on stroke-related costs for the first and second year after the incidence, we assumed that costs in all cost categories remained constant at the year-two value for the remaining life years.

#### Myocardial infarction

The DMCs and ICs related to MI (defined as “acute myocardial infarction” ICD-9-CM codes 410, 411) were derived from a paper by Mourad et al. [16]. Patients suffering from MI usually have several other diagnoses in the ischemic heart diseases group that can act as risk factors prior to the MI incidence (e.g. hypertension and hyperlipidaemia). However, Mourad et al. [16] did not disaggregate DMCs and ICs for MI, hypertension, or hyperlipidaemia, and thus it is likely that their MI costs overestimated the MI costs. To minimize such a possible overestimation, in this study we only assessed costs during the first year after an MI incident. The DNMCs were not accounted for due to a lack of existing Swedish data. Because the duration of MI was assumed to be one year, no discounting was applied.

#### Preterm birth

Because air pollution is associated with PTB occurring mainly after week 32, we calculated the costs of PTB for births occurring at weeks 33–36. Accordingly, the PTB calculations required literature sources that stratified impacts per gestational week.

The DMCs for PTB were calculated as the differences in costs between those born full term and those born preterm, according to the costs in the Swedish Cost Per Patient Database (ICD-10 codes O60.1A–060.1X, 060.3A–060.3X & P07.0–P07.3X) [2,7]. The Swedish Cost Per Patient Database aggregates labour costs associated with PTB over all gestational weeks, while US inpatient first-year cost data [2,8] show large differences in costs between those born before week 28 and those born after week 32, suggesting a large variation in average cost estimates in the Swedish data. Therefore, to estimate the costs for late PTBs, the US cost data and the Swedish gestational data were disaggregated in steps with the following assumptions. Swedish statistics show that around 1% of the Swedish births take place in week 22–32 and 3.8% in week 33–36 [29]. For the 1% we assumed that 20% occur before week 28 and 80% occur in week 28–32. We also assumed that the costs for births in week 32–36 are equal to those in week 33–36. With these assumptions we could calculate weighted costs for those born in week 33–36 by multiplying the relative distribution of costs per group from the US cost data [2,8] with the relative distribution of births from Swedish gestational data [29], the product of which we called the cost factor. The cost factor was calculated as follows.

$$C-factor_{bw.33-36}=\frac{br_{bw.33-36}}{\sum_{bw}br_{bw}}*\frac{USc_{cw.32-36}}{\sum_{cw}(br_{cw}*USc_{cw)}}$$ (2)

Where

*C-factor*_*bw*.*33-36*_ = the cost factor for those born in week 33–36,

*br* = the ratio of all births in Sweden per gestational age group,

*bw* = the PTB gestational age groups included in the birth data (<week.28, w.28–32, w.33–36),

*USc* = the surplus cost for PTB per gestational age group according to US statistics [28],

*cw* = PTB gestational age groups in cost data (<w.28, w.28–31, w.32–36).

The full-term age group was excluded in this C-factor calculation.

As percent of all Swedish births, the preterm births, equals 3.8%, 0.8%, and 0.2% for gestational week 33–36, 28–32, and <w.28, respectively. Correspondingly, of the preterm births 79%, 17%, and 4% for gestational week 33–-36, 28–-32, and <w.28, respectively. The corresponding US costs were available for the preterm births occurring on weeks 32–36, 28–31, and <28, corresponding to for US$ 8 960, 83 300, and 179 000, respectively. An assumption that the costs for preterm birth would be equal for preterm births for weeks 33–36 (for which the birth data from Sweden was available) and for 32–36 (for which the cost data was available from US). The sum product of [79%, 17%, 4%] * [8,960, 83,300, 179,000] equals 28 400. The cost factor value for those born week 33–36 is then 8 960/28 400 = 0.32.

Finally, the week 33–36 cost factor was multiplied by the cost difference for PTB derived from the Swedish Cost Per Patient Database [27] to obtain labour-related DMCs for PTB.

For IC estimates we included the costs related to assistance due to disability, lower education, lower income, and lower employment levels and increased numbers of days spent on sick-leave and early retirement [30–32]. Low cost estimates were based on gestational weeks 37–38 instead of weeks 33–36, and high cost estimates for costs related to average personal productivity losses for persons born preterm were from Vredin and Johansson [33]. See the S2 File for detailed cost categories, unit valuations, and durations.

### Informal costs

The InCs were estimated by applying a quality-adjusted life year (QALY) approach. For stroke and MI, the difference between the Swedish general populations’ average life quality and the outcome-specific QALY values was used to calculate the average loss of QALYs among stroke and MI survivors for their remaining life years (Table 2) [34–36]. Further, it is known that stroke survivors’ spouses report lower QALY values, and these were thus included in the calculations of InC for stroke [37]. To estimate QALY loss for PTB, we used descriptive statistics on the differences in prevalence of mental retardation, cerebral palsy, hearing loss, and vision impairment between those born at weeks 32–36 and those born full term [28], and we combined these with estimated QALY losses for the corresponding outcomes reported by Graig et al. [38].

**Table 2:** QALY weights for the general population and for incident cases of stroke, MI, and PTB and for spouses of stroke survivors.

	QALY weights	Disease duration in years	Reference
General population	0.91	NA	[34]
Stroke	0.61	11	[36]
Stroke survivors’ spouses	0.754*	11	[37]
MI	0.82	1	[35]
PTB-induced QALY loss	0.017**	10	[38]

*A reference value of 0.77 was used to calculate the yearly QALY loss of spouses to stroke patients [37].

**The middle value of the corresponding QALY loss associated with PTB was 0.017 per year for a duration of 10 years. QALY = quality-adjusted life year, MI = myocardial infarction, PTB = preterm birth, NA = not assessed.

The economic valuation of QALYs for the low and middle estimate was based on the threshold values applied by the Swedish Dental and Pharmaceutical Benefits Agency to evaluate the cost-effectiveness of drugs. The rule-of-thumb threshold is €_2016_ 53k (referred to in this study as the low estimate for one QALY). For the middle estimate we used €_2016_ 106k, which is one of the highest thresholds accepted for reimbursing a drug [39, 40]. For the high estimate we used a societal willingness to pay of €_2016_ 250k [41], which is based on a willingness-to-pay study and applied by the Swedish Transport Administration (STA) [42]. Later, Olofsson et al. published a scientific article based on the willingness-to-pay study conducted on commission from the STA, where the specific methods of valuation of a QALY with chained approaches is described in more detail [43].

## Results

### Stroke

The formal life-time costs of stroke varied from 66k up to 500k for the low and high values categorized based on remaining disability level after the incident (Table 3, S3 File). The middle values for DMC and DNMC were estimated to be 36k and 158k, respectively. ICs were highest for the low-cost estimates and lowest for those with high disability ranking among the stroke survivals. The value of loss of QALYs was estimated to correspond 119k up to 570k using low and high QALY valuations, respectively. The average NPVs of stroke were estimated to be 460k, where formal costs constituted approximately 50% of the total.

**Table 3:** Formal and informal costs for stroke, MI, and PTB stratified to low, middle, and high estimates discounted by 3.5% and the NPV as the sum of formal and informal costs.

**Formal Costs**									
	Stroke			MI			PTB		
	Low (MRS 0–2)	Middle estimate (All survivors)	High (MRS 5)	Low*	Middle estimate	High**	Low	Middle estimate	High
DMC									
Inpatient	-16 795	-29 706	-27 734	-9 179	-11 473	-13 768	-5 359	-6 699	-8039
Outpatient	-10 541	-6 724	-3 061	-1 133	-1 416	-1 699	NA	NA	NA
DNMC	-7 822	-158 270	-469 590	NA	NA	NA	NA	NA	NA
IC	-30 962	-30 095	-899	-2 055	-2 569	-3 083	- 8 171	-15 574	-20 804
Sum of formal costs	-66 119	-224 796	-501 285	-12 367	-15 458	- 18 550	-13 530	-22 273	-28 843
**Informal costs**									
Value of loss of QALY	-118 940	-237 880	-570 912	-4 040	-8 080	-19391	-5954	-11 909	-28 580
**NPV** ***	-185 059	-462 676	-1 072 197	-16 407	-23 538	-37 942	-19 484	- 34 182	-57 424

* Assumption of 20% lower costs from the middle estimate

**Assumption of 20% higher costs from the middle estimate

*** Sum of formal and informal costs calculated by summing the formal cost per category and valuation of loss of QALYs using low, middle, and high valuation

### MI

The formal costs for incident MI were estimated to vary between 12k and 18k, with a middle estimate of 15k (Table 3, S3 File). DNM costs corresponded to 84% of the formal costs, and 16% were estimated as ICs. The value of loss of QALYs was estimated to correspond to 8k when using the middle estimates. The average NPV of MI was estimated to correspond 24k, out of which formal costs contributed 65%.

### PTB

For PTB the average DMC corresponding to the medical resource use of the first life-year were estimated to be on average 7k (Table 3, S3 File). However, the average lifetime IC for PTB was estimated to be twice as large as the DMC. The value of loss of QALY using the central estimate was 12k. The average NPV of PTB was estimated to be 34k, out of which the formal costs made up 56%.

### Sensitivity analysis

When implementing the low monetary estimate of a QALY, the NPV per case was reduced by 20–30% for each health outcome (Table 3). In contrast, the high estimate of a QALY increased the total costs by 50–70%.

In the sensitivity analysis, we also varied the discount rate between 2% and 5% when estimating the NPVs for stroke and PTB (Table 4, S3 Table). For stroke, depending on the discount rate during the average disease duration, the average per-case total costs varied by ±6%, mainly due to effects of InCs on the NPV. The average per-case total costs for PTB were estimated to be 25% lower than in the middle analysis when assuming a 5% discount rate and to be 50% higher when assuming a 2% discount rate.

**Table 4:** Sensitivity analysis of varying discounts and QALY values for total cost estimates for stroke and PTB.

	Stroke			PTB		
	Low	Mid	High	Low	Mid	High
2% low QALY	-196 582	-324 024	-680 863	-26 358	-32 789	-50 796
2% mid QALY	- 364 234	-491 676	-848 515	-40 897	-47 328	-65 335
2% high QALY	-655 292	-782 734	-1 139 572	-51 561	-57991	-75 999
5% low QALY	-174 708	-286 054	-597 822	-15 639	-21 167	-36 647
5% mid QALY	-325 217	-436 563	-748 331	-21 283	-26 812	-42 291
5% high QALY	-588 389	-699 734	-1 011 502	-25 663	-31 192	-46 672

## Discussion

This study reports per-case costs of non-fatal stroke, MI, and PTB by including the formal costs and InCs in Sweden. Our results suggest that the socioeconomic costs of stroke, MI, and PTB are substantial and that InCs constitute on average 50% of the total costs. Even after discounting by varying rates, the total cost of stroke, MI, and PTB remained substantial. The results from this study exclude costs associated with fatal stroke and MI, as well as costs associated with very early PTB, and are thereby designed to facilitate application in already existing CBA tools assessing the monetary effects of air pollution interventions. Together with the ERF values presented in the introduction, these values provide a possibility to increase the number of air pollution impacts considered in CBAs.

Mortality is currently dominating the health economic burden in existing CBA tools. For example, in the ARP model that was used to support the latest National Emission Reduction Commitments (NEC) Directive of the European Union, the health economic burden of mortality corresponded to 66% of the total economic burden. The ARP model version that we compared our results to contains values for preterm fatality (estimated through Value of Statistical Life (VSL) or Value of a life year (VOLY)), preterm fatality of children, chronic bronchitis for adults and bronchitis for children, respiratory and cardiac hospital admissions, restricted activity days, asthma symptom days, and lost working days. Including these morbidity outcomes would alter the estimated proportion of the monetary burden attributable to mortality from the NEC value of 66% of total costs down to 40–60% given the uncertainty ranges.

When including the ERFs and the economic values of stroke, MI, and PTB in this version of the model we can estimate the economic benefits of a 1μg/m^3^ reduction in the annual average concentration of PM_2.5_ in Sweden. For a mortality VOLY valuation at €_2016_ 45k per life year, the comparison showed that the benefits of cleaner air would increase by some 12–74% if stroke, MI, and PTB are valued as in our low or high estimates respectively (or €_2016_ 440M–680M instead of €_2016_ 390M). For the same mortality value, the share of mortality of the total health effect value would be reduced from 66% to 51%. For a mortality value corresponding to a VSL of €_2016_ 3 150 000, the mortality share would be reduced from 93% to 88%.

The morbidity costs presented in this study highlight that the total economic burden due to air pollution is underestimated in current CBAs guiding European Union air pollution policy processes.

Our study estimates can also be applied to estimate the marginal benefits of reducing air pollution exposure for individual countries to meet air quality goals. For example, the Swedish Environmental Agency’s latest health impact assessment of air pollutants reports the average population-weighted PM_2.5_ exposure in 2019 to be 7.2 μg/m^3^ [44]. According to the report, 82% of the Swedish population is exposed to higher annual PM_2.5_ concentrations [44] than recommended by the World Health Organization’s latest Air Quality Guideline value of 5 μg/m^3^ [45]. If the annual average PM_2.5_ exposure is lowered by 1 μg/m^3^, then on average 178 incident MI cases and 616 incident stroke cases could be avoided, with a corresponding marginal economic benefit of €_2016_ 289M using this study’s middle estimates of NPVs.

### Formal costs

The results from this study are not directly comparable to earlier literature because we included indirect costs and excluded fatal outcomes for stroke and MI and preterm births earlier than 33 weeks gestational age. Regardless of these qualitative differences, our results are still sufficiently consistent with values from other studies. For example, the average cost reported per stroke patient in the EU in 2015 was €_2016_ 158k [7]. This is a little bit higher than annual costs reported by Lekander et al. [23], who reported an annual cost per stroke patient of €_2016_ 120k. Persson et al. [14] estimated the costs per stroke case to be €_2016_ 94k, which is comparable with this study’s low estimates corresponding to stroke survivals with low disability with a NPV of €_2016_ 66k–71k. In comparison with MI, the average cost of one ischemic heart disease incidence in 2015 was estimated to be €_2016_ 20k in Europe [7], which again compares well with this study’s average cost estimate of €_2016_ 24k. Previously published economic costs of PTB vary great. Trasande et al. [19] and Kim et al. [46] presented economic costs of €_2016_ 290k and €_2016_ 62k–188k per incidence of PTB, respectively. The US Committee on Understanding Premature Birth and Assuring Healthy Outcomes reported a unit value for PTB of €_2016_ 63k in the US, accounting for DMCs, IDMCs, and ICs and applying a 3% discount rate [28], which was twice as high as this study’s estimate. A likely explanation for the higher per-case cost estimate for PTB in previous research is that we accounted for costs for PTB that occurred during gestational weeks 33–36 in contrast to aggregated costs for all PTB cases regardless of gestational age. In the US, for example, PTB that occurs before gestational week 28 incurs 17-times higher costs than births in gestational week 32–36 [28]. Furthermore, previous estimates are based on US data, a country known to often have higher health care costs compared to Europe. As relevant examples, costs for normal delivery births and percutaneous transluminal coronary angioplasty have been reported to be 70% and 55% more expensive in the US than in Sweden [47]. The health care system in Sweden has universal access, which is associated with lower health care costs, whereas the US has a fragmented private insurance-based health care system, which is associated with higher health care costs. The socioeconomic cost for stroke and MI could also be used by other fields of research trying to quantify the economic burden of disease, but caution is needed if using the benefit transfer method to apply the economic cost estimates to other countries. Health care costs can vary due to the structure of the health care system, and lifetime levels of education and income are affected by, among other things, the degree of social mobility in the country of interest.

The use and value of discount rates is a debated praxis in the environmental economic literature, with renewed intensity after the Stern Review in 2006, mostly due to the ethical implications of the chosen values [48–50]. Following economic theory, an individual’s willingness to pay for goods received at the present time is greater than for goods received in the future. However, in environmental and health policy contexts, discounting the value of future benefits is controversial. If decision-makers are contemplating an intervention to reduce air pollution and are accounting for expected future health benefits, applying a discounting rate will reduce the total NPV of the health benefits. For example, applying a 5% discount rate on our results leads to greater reductions in InCs compared to formal costs. A declining discount rate has been suggested as a method for evaluating the economic benefits of health interventions with immediately occurring implementation costs but with benefits that are expected to occur in the far future [51, 52]. While this discussion is of interest, there is to our knowledge a lack of empirical studies that have tested the effect of discounting of health effects on decision-making outcomes.

As a strength of this study, total cost estimates for stroke were based on disaggregated cost data for stroke survivors, which created good premises to estimate lifetime costs taking into consideration the large variation in costs between different mRS strata [23]. This study also included the losses of QALY to be able to account for informal costs, which are often excluded in environmental economics due to theoretical differences in the methods for valuing a QALY (threshold vs. willingness-to-pay). In the Swedish context, the health economics evaluations rely on threshold values to assess cost-effectiveness in terms of QALY’s gained, but this method has little empirical support. However, environmental economic theory prefers a QALY valuation based on willingness-to-pay studies. Nevertheless, this study is the first to quantify the InCs, which were estimated to corresponded to approximately 50% of the total costs, illustrating the possible depth of underestimation of socioeconomic costs due to stroke, MI, and PTB. Secondly, we demonstrated the uncertainty ranges based on the monetary values applied in praxis to value a QALY, therefore suggesting a possible direction for developing more interdisciplinary valuation methods applicable to both health and environmental economic disciplines. Additionally, extension of the theoretical framework is warranted to serve as a foundation for future empirical studies trying to estimate the willingness-to-pay value for a QALY that could be applicable in both fields of research.

A shortcoming of this study is that all costs were not available for all the recommended cost categories. Stroke and MI socioeconomic cost estimates are also subject to inconsistency related to source of data reporting [16, 23]. Most prominently, the costs for control groups are often not reported, which leads to the risk of double-counting some fraction of the economic costs for the outcomes in question. Thus, a potential overestimation of socioeconomic costs associated with morbidity outcomes should be considered. However, if we compare the average DMC of 65-year-olds in the Swedish county of Västra Götaland with the DMC of MI patients, the latter has five times the average DMC compared to health care consumption for an average person [53]. This supports the notion that both stroke and MI incidence result in a substantial increase in DMC and suggests that the overestimation induced by the absence of control groups in the source data is likely to be small.

With respect to PTB, the outpatient DMC and DNMC items were not covered in the Swedish data or literature and were therefore excluded from the main analysis. To check the potential importance of these items, we used cost estimates from the US as a proxy, which resulted in a 9% higher central estimate compared to our main analysis (SM10, SM11). The difference from the central estimate in the main analysis was not very large, which indicates that our central estimate included most of the relevant costs.

The PTB InCs were probably underestimated because the parents of a PTB probably suffer a loss of QALY compared to parents to a normal gestational age (38–42 weeks) similarly to caregivers of stroke patients. However, there is a need to quantify the estimated utility losses among the PTB parents in future studies to also be able to account for these in economic evaluation studies.

### Recommendations

Future research should focus on acquiring more relevant data, especially for MI and the quality-of-life effects of PTB. Research should also focus on continuing the economic valuation of the other health outcomes related to air pollution exposure [3]. Continuing the valuation of morbidity outcomes of air pollution exposure is important because it is likely that these outcomes affect the welfare-maximising level of pollution as analysed in the cost-benefit analyses currently used to support air pollution policies in Europe.

## Conclusion

This study reports socioeconomic cost estimates for stroke, MI, and PTB to facilitate the upgrade process of CBAs attempting to monetize the adverse health effects of air pollution. The valuation method proposed by Hodek et al. [22] and the data available in Sweden were sufficient to establish reliable cost estimates for this aim. Other CBA research fields such as climate economics can use the monetary values reported here for stroke, MI, and PTB as input values as well. If including formal and informal welfare costs of stroke, MI, and PTB in the group of already established welfare costs of air pollution (such as loss of preterm fatality, chronic bronchitis, etc.), the quantified benefits of cleaner air would increase substantially in the Swedish case. The inclusion of monetized values of stroke, MI, and PTB is important prior to future analyses of the costs and benefits of air pollution emission control.

## Supporting information

S1 File Search terms applied and results of literature search in PubMed to retrieve cost-of-illness studies for stroke and MI. (PDF)

S2 File Data on occurrence, duration, and indirect costs associated with preterm birth. (PDF)

S3 File Disaggregated costs-per case of stroke, MI and PTB and sensitivity analyses. (XLSX)
