# Supplementary material for: Socioeconomic per-case costs of stroke, myocardial infarction, and preterm birth attributable to air pollution in Sweden
Source: PLoS One. 2024 Jan 11;19(1):e0290766. doi: 10.1371/journal.pone.0290766 (PMC10783732; doi:10.1371/journal.pone.0290766)
Supplement: S1 File — (PDF) [file pone.0290766.s001.pdf]

Supporting information File 1.

Search terms applied and results of literature search in PubMed to retrive cost-of-illness studies for stroke and MI.

Health outcome	Search Term	Results in Pubmed	Notes
Stroke	(("cost of disease" OR "societal cost" OR "cost of illness")) AND stroke AND “Sweden”))	51	Removed:<br>34 since not economic studies,<br>15 removed since not meeting the inclusion criterias
MI	(("cost of disease" OR "societal cost" OR "cost of illness")) AND “ischemic heart disease” AND “Sweden”))	7	Removed:<br>6 removed<br>5 not economic studies,<br>1 removed since not MI
