# Supplementary material for: Socioeconomic per-case costs of stroke, myocardial infarction, and preterm birth attributable to air pollution in Sweden
Source: PLoS One. 2024 Jan 11;19(1):e0290766. doi: 10.1371/journal.pone.0290766 (PMC10783732; doi:10.1371/journal.pone.0290766)
Supplement: S2 File — (PDF) [file pone.0290766.s002.pdf]

Supporting information File 2.

Data on occurance and duration of preterm birth (PTB) cost categories (Table SM1) and detailed data for PTB indirect cost (IC) estimates (Tables SM2, 3).

Table SM 1: Health and social impacts included in the monetization of preterm birth, their assumed age of occurrence, and the assumed duration of the impact (Lindström et al., 2007 & Statistics Sweden 2016)

Cost category and sub-category	***Age of occurrence (years)***	***Duration (years)***
DMC – Visit to physician	0 (child birth)	0
DNMC – Outpatient/Home help	23*	60**
IC – Income losses: education	23*	41**
IC – Income losses: employment	23*	41**
IC – Missed working days: sick leave	23*	41**
IC – Income losses: sick retiree	23*	41**

*Assumption based on the first year for which there is data (Lindström et al., 2007, Husby et al., 2016)

** Assumption based on retirement at 65 years of age and an average lifetime of 82 years (National Board of Health and Welfare 2015).

Table SM 2: IC – Income losses: education. 2002 education level as a function of gestational age at birth for persons aged 23–29 years (Lindström et al., 2007), salaries as a function of education level (Statistics Sweden 2016) and corresponding income losses from education effects of being born preterm

	Gestational age, week, n, (% of n)		
	Week 33–36	Week 37–38	Week 39–41
	N = 19 166	N = 68 541	N = 431 656
Education level			
Missing or <9 years	161 (0.8)	386 (0.6)	1 583 (0.4)
Basic 9 years	2 128 (11.1)	6 902 (10.1)	38 546 (8.9)
10–11 years	2 437 (12.7)	7 922 (11.6)	51 596 (12.0)
12–13 years	7 638 (39.9)	27 157 (39.6)	168 131 (39.0)

Postsecondary ≥ 14 years	6 802 (35.5)	26 174 (38.2)	171 800 (39.8)
Salary per month incl. tax (PPP€$_{2016}$)			
Missing or <9 years	1 992		
Basic 9 years	2 190		
10–11 years	2 339		
12–13 years	2 280		
Postsecondary ≥ 14 years	3 158		
Weighted yearly salary per person given 72.5%* employment rate (PPP€$_{2016}$)			
	22 505		22 876
Income loss per year (week 33-36 vs. week 39-41) (PPP€$_{2016}$)			
	-372		

*The employment rate for those born in week 33-36 (Table SM3)

Table SM 3: IC – Income losses: employment, missed working days, and sick retirees. The 2002 employment levels, sick allowance levels, and sick retiree levels as a function of gestational age at birth for persons age 23-29 years (Lindström et al., 2007). Income losses based on salaries per month as presented in Table SM2 (Statistics Sweden 2016). Missed working days are represented by the percentage of persons receiving sick-leave allowance.

	Gestational age, week, n, (% of n)		
	Week 33–36	Week 37–38	Week 39–41
	N = 19 166	N = 68 541	N = 431 656
Employment in Nov. 2002	13 903 (72.5)	49 809 (72.7)	319 729 (74.1)
Sick-leave allowance	1 316 (6.9)	4267 (6.2)	25 331 (5.9)
Sick retiree	468 (2.4)	1 162 (1.7)	5702 (1.3)
Income loss per year (week 33-36 vs week 39-41) (PPP€$_{2016}$)			
Employment in Nov. 2002	-497		
Missed working days	-310		
Sick retiree	-341		

References to supplementary material 2

Lindström, K., et al., Preterm infants as young adults: a Swedish national cohort study. Pediatrics, 2007. 120(1): p. 70-7.

Husby, I.M., et al., Long-term follow-up of mental health, health-related quality of life and associations with motor skills in young adults born preterm with very low birth weight. Health Qual Life Outcomes, 2016. 14: p. 56.

National Board of Health and Welfare (2015). Graviditeter, förlossningar och nyfödda barn, Medicinska födelseregistret 1973–2014, Assisterad befruktning 1991–2013.

Statistics Sweden (2016). Statistical Database, Average monthly salary and level of education (SUN 2000 classification) (1991 - 2015) Average population 16-74 years per education level, age, and gender (1985 - 2015), http://www.statistikdatabasen.scb.se/pxweb/en/ssd/?rxid=6ea971a7-8a94-4376-875e-9de188897068
